# Risk factors for late (28+ weeks’ gestation) stillbirth in the United States, 2014–2015

**DOI:** 10.1371/journal.pone.0289405

**Published:** 2023-08-30

**Authors:** Darren Tanner, Sushama Murthy, Juan M. Lavista Ferres, Jan-Marino Ramirez, Edwin A. Mitchell

**Affiliations:** 1 AI for Good Research Lab, Microsoft Corporation, Redmond, WA, United States of America; 2 Center for Integrative Brain Research, Seattle Children’s Research Institute, Seattle, WA, United States of America; 3 Departments of Neurological Surgery and Pediatrics, School of Medicine, University of Washington, Seattle, WA, United States of America; 4 Department of Paediatrics, Child and Youth Health, The University of Auckland, Auckland, New Zealand; University of Tennessee Knoxville, UNITED STATES

## Abstract

**Background:**

In the United States (US) late stillbirth (at 28 weeks or more of gestation) occurs in 3/1000 births.

**Aim:**

We examined risk factors for late stillbirth with the specific goal of identifying modifiable factors that contribute substantially to stillbirth burden.

**Setting:**

All singleton births in the US for 2014–2015.

**Methods:**

We used a retrospective population-based design to assess the effects of multiple factors on the risk of late stillbirth in the US. Data were drawn from the US Centers for Disease Control and Prevention live birth and fetal death data files.

**Results:**

There were 6,732,157 live and 18,334 stillbirths available for analysis (late stillbirth rate = 2.72/1000 births). The importance of sociodemographic determinants was shown by higher risks for Black and Native Hawaiian and Other Pacific Islander mothers compared with White mothers, mothers with low educational attainment, and older mothers. Among modifiable risk factors, delayed/absent prenatal care, diabetes, hypertension, and maternal smoking were associated with increased risk, though they accounted for only 3–6% of stillbirths each. Two factors accounted for the largest proportion of late stillbirths: high maternal body mass index (BMI; 15%) and infants who were small for gestational age (38%). Participation in the supplemental nutrition for women, infants and children program was associated with a 28% reduction in overall stillbirth burden.

**Conclusions:**

This study provides population-based evidence for stillbirth risk in the US. A high proportion of late stillbirths was associated with high maternal BMI and small for gestational age, whereas participation in supplemental nutrition programs was associated with a large reduction in stillbirth burden. Addressing obesity and fetal growth restriction, as well as broadening participation in nutritional supplementation programs could reduce late stillbirths.

## Introduction

Fetal loss is the death of the fetus during pregnancy or labor. In the United States (US) a death that occurs prior to 20 weeks’ gestation is usually classified as either a spontaneous miscarriage or termination of pregnancy; those occurring after 20 weeks constitute a stillbirth. Because of differences in reporting and definitions of stillbirth across countries, the World Health Organization (WHO) uses fetal deaths from 28 weeks’ gestation for international comparisons. Using this definition more than 2.6 million stillbirths occur per year worldwide, most of which are in low and middle-income countries where they are frequently related to lack of access to adequate care in pregnancy and labor [1].

In high-income countries, stillbirth rates declined markedly between 1940 and 1990 due largely to improvements in maternity care [2]. However, this decline has slowed: between 1990 and 2008, late stillbirth rates declined by only 14% across 12 high-income countries [3]. Since then, the decline in late stillbirth rates in the US has further slowed: rates plateaued between 2006 (2.97/1000 births) and 2012 (2.96/1000) [4], though the rate had declined slightly by 2019 (2.73/1000), although at least part of this reported decrease is due a change in definition used to measure gestational age [5].

Rates also vary between high-income countries. In the US in 2015 the late stillbirth rate was 3.0/1000 births compared with Iceland 1.3/1000, Denmark 1.7/1000, The Netherlands 1.8/1000, Norway 2.2/1000 and UK 2.9/1000 [6]. This suggests there is considerable room for improvement in the US.

Known risk factors for stillbirth in high-income countries include mothers who are nulliparous, have a plural pregnancy, are older, belong to ethnic minority groups, experience socioeconomic disadvantage, have high BMI, smoke tobacco, have delayed or absent prenatal care, have diabetes, and/or have hypertension [7]. However, most of the studies reporting these factors have defined stillbirth as occurring after 20 weeks’ gestation rather than the WHO’s 28 weeks’ definition. The Stillbirth Collaborative Research Network has shown that the cause of stillbirth varies by gestational age, with relative rates of causes differing between those that occur between 20–27 weeks of gestation (“early” stillbirth) and those occurring at 28 completed weeks of gestation or more (“late” stillbirth): obstetric complications were the cause in 39.8% of early but only 17.4% in late stillbirths; infections were implicated in 16.9% of early but only 8.7% of late stillbirths; placental problems were implicated in 20.9% of early, but 25.7% of late stillbirths; and unknown causes were implicated in 20.5% of early, but 27.2% of late stillbirths [8].

Given that the causes of stillbirth differ by gestational age, it is conceivable that risk factors may also vary with gestational age; characterizing only the broader age range may miss this important point and may therefore also hinder our understanding of the relative contribution of risk factors at particular gestational ages. For example, Flenady and colleagues’ [3] meta-analysis of risk factors for stillbirth in high-income countries noted that, of the 96 studies they considered, 62 studies considered gestational periods beginning prior to 28 weeks and an additional 4 used gestational cutoffs beginning later than 28 weeks. Additional population-based work focusing on late stillbirth as defined by the WHO is therefore needed. Moreover, at gestational ages of 28 weeks and beyond there could be a high chance of intact survival had at-risk babies been identified, treated, or delivered alive [9]. Gaining a clearer understanding of factors related this gestational period is therefore of high importance.

Additionally, there are variables that can be explored that have not been considered in relation to stillbirth that can be examined at the national level, such as nutritional supplementation for mothers. Nutritional supplementation has shown benefits for improving neonatal and child health outcomes [10–15], though to our knowledge no US-level population-based research has investigated whether supplementation programs are associated with reduced stillbirth rates. Identifying whether there may be a potential association between nutritional support and reduced stillbirth is thus an important avenue for research. We view this as hypothesis generating research.

The aim of this study was therefore to examine risk factors for stillbirth in the US, specifically focusing on gestational ages for stillbirth as defined by the WHO (late stillbirth: 28+ weeks’ gestation). We provide updated population-based risk estimates for potentially modifiable factors that contribute substantially to late stillbirths, and we also quantify the proportion of overall late stillbirth burden that are associated with each of these factors in the US. This also includes providing an initial US population-level assessment for the potential role of participation in maternal nutritional supplementation programs in reducing stillbirth risk in the US.

## Methods

### Data source and study population

We used a retrospective population-based design to assess the effects of multiple factors on risk of late stillbirth in the US. Data were drawn from the US Centers for Disease Control and Prevention (CDC) live birth data and fetal death data files for the years 2014 and 2015 [16,17]. Stillbirths are reported by most states for deaths occurring at 20 or more weeks of gestation or at least 350 grams of delivery weight. Data from the respective files include all live and stillbirth events reported in the US for 2014–2015, with information for 7,986,908 live and 104,362 stillbirths over the study period.

**Fig 1:**
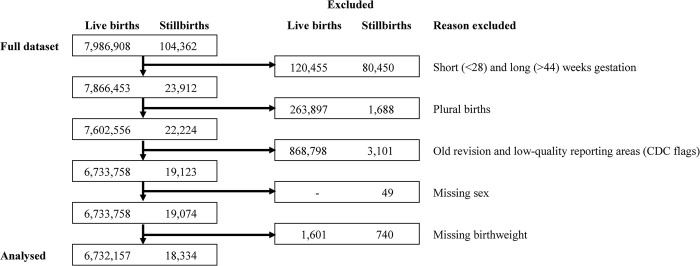
Flow diagram of births and reasons for exclusion.

As the cases of interest are late stillbirths, we excluded all live births and stillbirths before 28 and after 44 weeks of gestation. Gestational age in completed weeks was determined by the CDC using obstetric estimate of gestation at delivery. We additionally excluded cases involving plural births and those with missing sex or birthweight. The CDC provides reporting flags indicating data that were either collected using unrevised 1989 reporting version of the US Standard Report of Fetal Death or US Standard Certificate of Live Birth, as opposed to the current revised 2003 version, or from areas that the CDC determines have low reporting quality. Data from the unrevised forms and low-quality data were excluded based on the CDC flags. Case flow information is provided in Fig 1. We used publicly available data, so IRB approval was not required.

### Explanatory variables

Only risk factors common to the live birth and fetal death files were included in our analyses. The following factors were examined, with the exact combination of factors dependent on the specific analysis (see below): maternal age (categorized <15 years, 15–19, 20–24, 25–29, 30–34, 35–39, 40–44, 45+); paternal age (<15, 15–19, 20–24, 25–29, 30–34, 35–39, 40–44, 45–49, 50+, unknown or not stated), self-reported maternal educational attainment (8th grade or less, 9th-12th grade, high school graduate or GED, some college, associate degree, bachelor’s degree, master’s degree, doctorate or doctoral-level professional degree, unknown or not stated); self-reported maternal race (White, Black, American Indian/Alaska Native, Asian, Native Hawaiian and Other Pacific Islander, more than one race, unknown or not stated); self-reported maternal Hispanic ethnic origin (non-Hispanic, Mexican, Puerto Rican, Cuban, Central and South American, other and unknown Hispanic origin, unknown or not stated); maternal place of birth (nativity; born in the US (50 states), born outside the US (incl. territories), unknown or not stated); prepregnancy maternal BMI (underweight (<18.5 kg/m^2^), normal (18.5–24.9), overweight (25.0–29.9), obesity I (30.0–34.9), obesity II (35.0–39.9), obesity III (40.0+), unknown or not stated); self-reported participation in the supplemental nutrition for Women, Infants, and Children program (WIC; no, yes, unknown or not stated); self-reported maternal smoking status (did not smoke in the three months prior to or during pregnancy (non-smoker), smoked before and during pregnancy (continued smoking), smoked before pregnancy but stopped by the first trimester (quit smoking), did not smoke before pregnancy but reported smoking at some point during pregnancy (started smoking), unknown or not stated); diabetes (no diabetes diagnosis, prepregnancy diabetes, gestational diabetes, unknown or not stated); hypertension (no diagnosis of hypertension, prepregnancy hypertension only, gestational hypertension only (which includes pregnancy-induced hypertension and pre-eclampsia, as these are included as a single category on the US Standard Report of Fetal Death and US Standard Certificate of Live Birth), eclampsia, unknown or not stated); use of infertility treatment (no, yes, unknown or not stated); number of previous live births (no previous live birth, 1, 2, 3, 4, 5 6, 7+); interval since last live birth (4–11 months, 12–17, 18–23, 24–35, 36–47, 48–59, 60–71, 72+, unknown or not stated); timing of start of prenatal care (1^st^-3^rd^ month, 4^th^-6^th^, 7^th^-final month, no prenatal care, unknown or not stated); whether the mother had undergone a cesarean section for a prior pregnancy (no, yes, unknown or not stated); whether any of the mother’s prior live births were now dead (prior live birth now dead; no, yes, unknown or not stated); sex of fetus (female, male); and fetal growth (small for gestational age (SGA, ≤10 centile), appropriate for gestational age (AGA, >10 - <90 centile), large for gestational age (LGA, ≥90 centile)), which was estimated using the Gestation Related Optimal Weight standard (Customised Centile Calculator GROW v8.0.6.1) [18,19]. GROW centiles reflect estimated optimal fetal development weight at birth by gestation age, adjusted for maternal country of origin/race, maternal height and weight, parity, infant sex, gestational duration, and outcome (live birth or fetal death). For GROW, the following variable imputation methods were used as implemented in the GROW calculation software: no centile was calculated if birthweight was less than 150g or greater than 8000g or if gestation period was less than 140 days or greater than 308 days; if height and/or weight were missing, the average for the ethnicity/country of origin was used; if either of these values and ethnicity/country of origin were missing, the global average were used; if parity was missing, the value 1 was used [18].

### Statistical analysis

To understand the relationship between multiple factors and the risk for stillbirth, we fit a series of generalized linear models (GLMs) in R [20]. Each analysis estimated relative risk ratios (RRs) for stillbirth using a GLM with a quasi-Poisson distribution to adjust for over- or under-dispersion in variance and used a log link function. Univariate RRs were estimated for each risk factor individually, and adjusted risk ratios (aRRs) were estimated from multivariate models.

Our main analyses focused on two subsets of observations. The first subset included all observations after the exclusions described above (Fig 1). Because of potential complex causative and correlational relationships among the risk factors under consideration, we fit a series of three stratified GLMs on this subset. These GLMs did not consider interval since last live birth, prior cesarean, and prior live birth now dead, as these risk factors would be irrelevant for those mothers in the dataset with no prior live birth. The stratified approach was chosen to mitigate risk of overadjustment or collider bias for reported aRRs due to the complex relationships between the risk factors under study [21–25]. Some previous research on stillbirth and other adverse perinatal outcomes has additionally adjusted for gestational age in some analyses; however, as adjusting for gestational age has been shown to be problematic and potentially give biased estimates for other exposures due to mediation and collision [26–28], we have not done so in the present work. Note that interpretation of all adjusted coefficients for all models is subject to the additional assumption that stillbirth and any potential mediating variables have no unmeasured confounding [24].

The first GLM considered only sociodemographic variables (maternal age, paternal age, maternal education, maternal race, maternal Hispanic origin, maternal nativity), as these are social determinants of health. The second GLM focused on medical/obstetric factors and supplemental nutrition (number of previous live births, previous cesarean, infertility treatment, diabetes, hypertension, maternal smoking, maternal prepregnancy BMI, timing of prenatal care, infant sex, WIC), while adjusting for the sociodemographic factors included in the first model. For this model we only report the coefficients associated with the medical/obstetric factors and WIC, after adjustment for sociodemographic factors. The third model focused on risk associated with fetal growth (SGA and LGA), after adjusting for all factors from the previous two models. From this model we only report the coefficients for SGA and LGA, which reflect the residual relative risk estimates after controlling for all other factors. Note that all reported aRR coefficients for all models reflect partial (not total) effects after controlling for other variables in the reported model, as well as variables from previous models when relevant. Adjustment variables for each model are reported in table legends.

The second subset included observations for those mothers with at least one prior live birth and focused on the risk factors relevant to this sub-group: interval since the last live birth, prior cesarean, and having a prior live birth now dead. We fit two separate GLMs in a similar fashion to the above analyses. The first GLM included all risk factors except GROW and prior live birth now dead; from this model we report risk estimates for interval since last live birth and prior cesarean. The second model included all risk factors; from this model we report the risk estimate for prior live birth now dead, which reflects the remaining residual risk for mothers who have had this prior bad outcome after controlling for other available sociodemographic, medical, and obstetric risk factors associated with the present pregnancy.

As risk factors may differ between term births (37 to 41 completed weeks’ gestation) and pre-(<37 weeks)/post-term (≥42 weeks) births, we conducted a sensitivity analysis to exclude potential impacts of pre- and post-term births on risk estimates. This analysis focused only on term pregnancies by limiting the data to those births occurring between the 37^th^ and 41^st^ weeks of pregnancy (inclusive). We fit three models using the same stratified approach we used for the analyses with all observations, described above.

In the multivariate models, overlap in the unknown/not-stated observations across a subset of obstetric/medical risk factors led to model singularities and inestimable coefficients for the unknown/not stated level for these risk factors. These coefficients are listed as undefined in results tables. For each model, model fit was assessed using the area under the receiver operator characteristic curve (AUC). AUC quantifies the ability of a model to discriminate between stillbirths and live births and allows comparison across models both within this study as well as between studies. An AUC of 0.5 reflects random chance discrimination and an AUC of 1.0 reflects perfect discrimination.

### Population attributable fractions

To assess the contribution of potentially modifiable risk factors to overall stillbirth burden, we calculated population attributable fractions (PAFs) for maternal BMI, maternal smoking, receipt of WIC support, receipt and timing of prenatal care, diabetes, hypertension, and GROW (SGA/LGA). PAF point estimates for each level of these variables were calculated from aRRs estimated by the models fit on all observations described above, thus adjusting for confounding. PAFs for GROW and the other risk factors calculated from the separate respective models; 95% confidence intervals (CIs) for PAF estimates were calculated using the stratified bootstrap method with 500 iterations to form the empirical PAF distribution then and taking the 0.025 and 0.975 quantiles across iterations [29–31].

## Results

**Fig 2:**
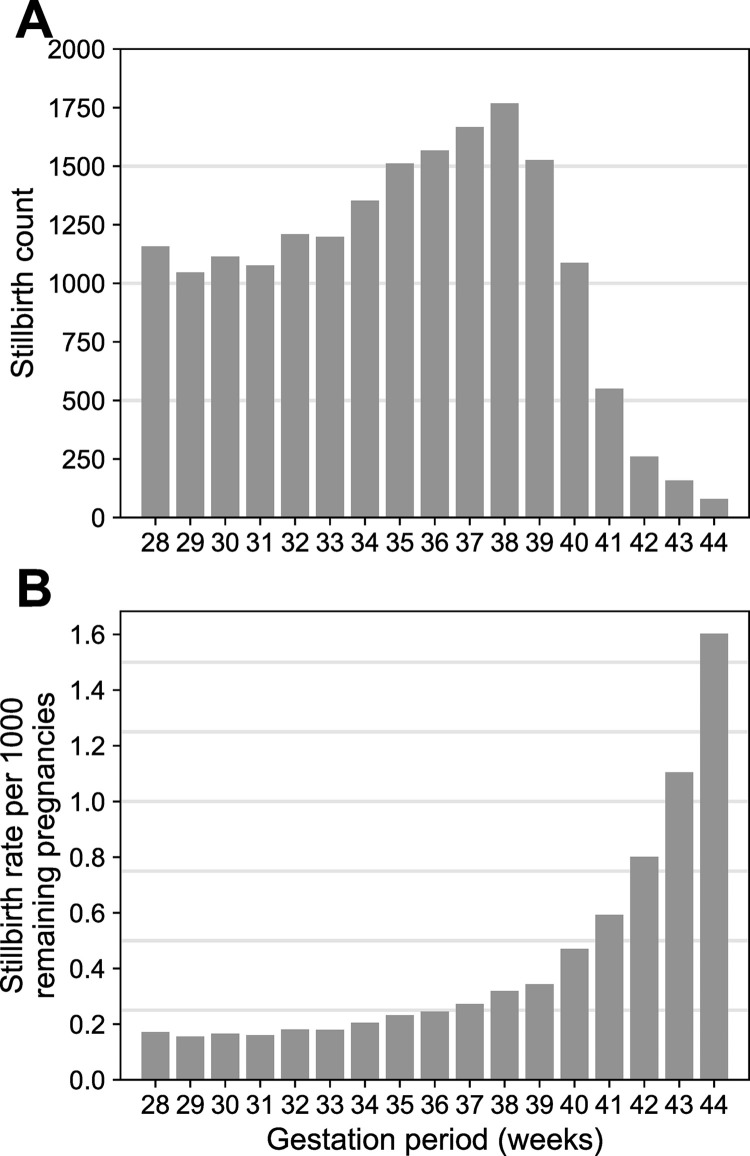
Stillbirth counts and risk by gestation period. Panel A shows the number of stillbirths by week of gestation. Panel B shows the risk of stillbirth per 1,000 remaining pregnancies by week of gestation.

Within our cohort there were 6,732,157 live and 18,334 stillbirths for analysis (late stillbirth rate for singleton births = 2.72/1000). Fig 2 shows the number of stillbirths and the risk of stillbirth per 1,000 remaining pregnancies by week of gestation. The number of stillbirths does not vary much until after 40 weeks when the absolute count drops considerably. In contrast, the risk of stillbirth for fetuses remaining in utero increases rapidly beginning in the 40^th^ week indicating substantially increasing stillbirth risk from 40 weeks of gestation.

### Risk models for all observations

As described above, we fit three separate GLMs. The first focused on sociodemographic factors. The second focused on medical/obstetric factors, except for GROW, and on nutritional supplementation, while controlling for sociodemographic factors; only coefficients for medical/obstetric factors and nutritional supplementation are reported for this model. The third focused on GROW, while controlling for all previous variables (sociodemographics, medical/obstetric factors, nutritional supplementation); only the coefficient for GROW is reported for this final model.

Table 1 shows results from the first (sociodemographic) model. As the univariate and adjusted models were similar for most variables, we highlight the adjusted findings. The importance of sociodemographic determinants is reflected in higher risks for Black and Native Hawaiian and Other Pacific Islander mothers compared with White mothers. When compared with mothers of non-Hispanic ethnicity, mothers of Puerto Rican Hispanic ethnicity had lower risk of late stillbirth. Maternal education showed a gradient of risk of stillbirth from the lowest level of educational achievement through to the highest. Mothers over 35 years of age were at increased risk of stillbirth, while the youngest mothers, although at increased risk in the unadjusted analysis, were at significantly lower risk after considering other sociodemographic factors. AUC of this model was 0.66.

**Table 1:** Unadjusted and adjusted relative risk ratios (aRR) over all observations for sociodemographic factors.

Variable	Live birth N	Stillbirth N	Stillbirth rate per 1000	RR (95% CI)	*Adjusted RR (95% CI)
**Mother’s race**					
White	5,051,632	12,147	2.40	ref.	ref.
Black	993,440	4,666	4.67	**1.95 (1.88, 2.02)**	**1.54 (1.49, 1.60)**
American Indian/Alaska Native	72,842	242	3.31	**1.38 (1.22, 1.57)**	1.10 (0.97, 1.26)
Asian	436,663	811	1.85	**0.77 (0.72, 0.83)**	0.97 (0.90, 1.05)
Native Hawaiian and Other Pacific Islander	19,752	112	5.64	**2.35 (1.95, 2.83)**	**1.67 (1.38, 2.01)**
More than one race	154,557	314	2.03	**0.85 (0.76, 0.95)**	**0.81 (0.73, 0.91)**
Unknown or not stated	3,271	42			
**Mother’s Hispanic origin**					
Non-Hispanic	5,037,703	14,155	2.80	ref.	ref.
Mexican	1,022,781	2,663	2.60	**0.93 (0.89, 0.97)**	1.02 (0.98, 1.07)
Puerto Rican	96,633	217	2.24	**0.80 (0.70, 0.91)**	**0.74 (0.64, 0.84)**
Cuban	36,768	74	2.01	**0.72 (0.57, 0.90)**	0.94 (0.75, 1.18)
Central and South American	228,111	522	2.28	**0.81 (0.75, 0.89)**	0.95 (0.87, 1.05)
Other and unknown Hispanic origin	258,577	396	1.53	**0.55 (0.49, 0.60)**	**0.55 (0.50, 0.61)**
Unknown or not stated	51,584	307			
**Mother’s age**					
Under 15	4,493	20	4.43	**1.74 (1.12, 2.70)**	0.70 (0.45, 1.10)
15–19	415,378	1,327	3.18	**1.25 (1.18, 1.33)**	**0.79 (0.73, 0.84)**
20–24	1,488,862	4,178	2.80	**1.10 (1.06, 1.15)**	**0.86 (0.82, 0.90)**
25–29	1,952,387	4,977	2.54	ref.	ref.
30–34	1,817,646	4,389	2.41	**0.95 (0.91, 0.99)**	**1.11 (1.06, 1.16)**
35–39	858,862	2,497	2.90	**1.14 (1.09, 1.20)**	**1.33 (1.26, 1.41)**
40–44	182,357	865	4.72	**1.86 (1.73, 2.00)**	**1.99 (1.83, 2.15)**
45+	12,172	81	6.61	**2.60 (2.09, 3.24)**	**2.42 (1.93, 3.03)**
**Mother’s education**					
8th grade or less	244,738	867	3.53	**1.96 (1.81, 2.12)**	**2.29 (2.10, 2.49)**
9th-12th grade	756,080	2,450	3.23	**1.79 (1.69, 1.90)**	**1.75 (1.65, 1.87)**
High school graduate or GED	1,676,774	5,411	3.22	**1.79 (1.70, 1.87)**	**1.72 (1.63, 1.81)**
Some college	1,425,940	3,387	2.37	**1.32 (1.25, 1.39)**	**1.25 (1.19, 1.32)**
Associate degree	538,591	1,232	2.28	**1.27 (1.18, 1.36)**	**1.23 (1.15, 1.32)**
Bachelor’s degree	1,278,414	2,307	1.80	ref.	ref.
Master’s degree	560,897	893	1.59	**0.88 (0.82, 0.95)**	**0.86 (0.79, 0.93)**
Doctorate or doctoral-level professional degree	161,903	232	1.43	**0.79 (0.69, 0.91)**	**0.77 (0.67, 0.88)**
Unknown or not stated	88,820	1,555			
**Mother’s nativity**					
Born in the US (50 states)	5,183,569	14,012	2.70	ref.	ref.
Born outside the US (incl. territories)	1,534,149	3,088	2.01	**0.75 (0.72, 0.77)**	**0.64 (0.61, 0.67)**
Unknown or not stated	14,439	1,234			
**Father’s age**					
Under 15	467	3	6.38	2.82 (0.91, 8.74)	2.43 (0.77, 7.67)
15–19	143,132	462	3.22	**1.42 (1.29, 1.57)**	**1.35 (1.21, 1.51)**
20–24	843,816	2,242	2.65	**1.17 (1.11, 1.24)**	**1.13 (1.07, 1.20)**
25–29	1,483,126	3,361	2.26	ref.	ref.
30–34	1,729,820	3,693	2.13	**0.94 (0.90, 0.99)**	0.96 (0.91, 1.00)
35–39	1,065,388	2,459	2.30	1.02 (0.97, 1.07)	0.95 (0.90, 1.01)
40–44	435,349	1,192	2.73	**1.21 (1.13, 1.29)**	0.98 (0.91, 1.06)
45–49	146,077	491	3.35	**1.48 (1.35, 1.63)**	1.08 (0.97, 1.19)
50+	68,156	295	4.31	**1.91 (1.69, 2.15)**	**1.31 (1.16, 1.49)**
Unknown or not stated	816,826	4,136			

Number of live births and late stillbirths, late stillbirth rate per 1000 births, and the unadjusted and adjusted relative risks (RRs) and their 95% confidence intervals (CI) for sociodemographic variables. Numbers in bold indicate category is significantly different from the reference category (ref). In this and all subsequent tables, we report counts but not rates and risk ratios for Unknown or not stated factor levels.

*Adjusted RRs are adjusted for all variables in the table.

**Fig 3:**
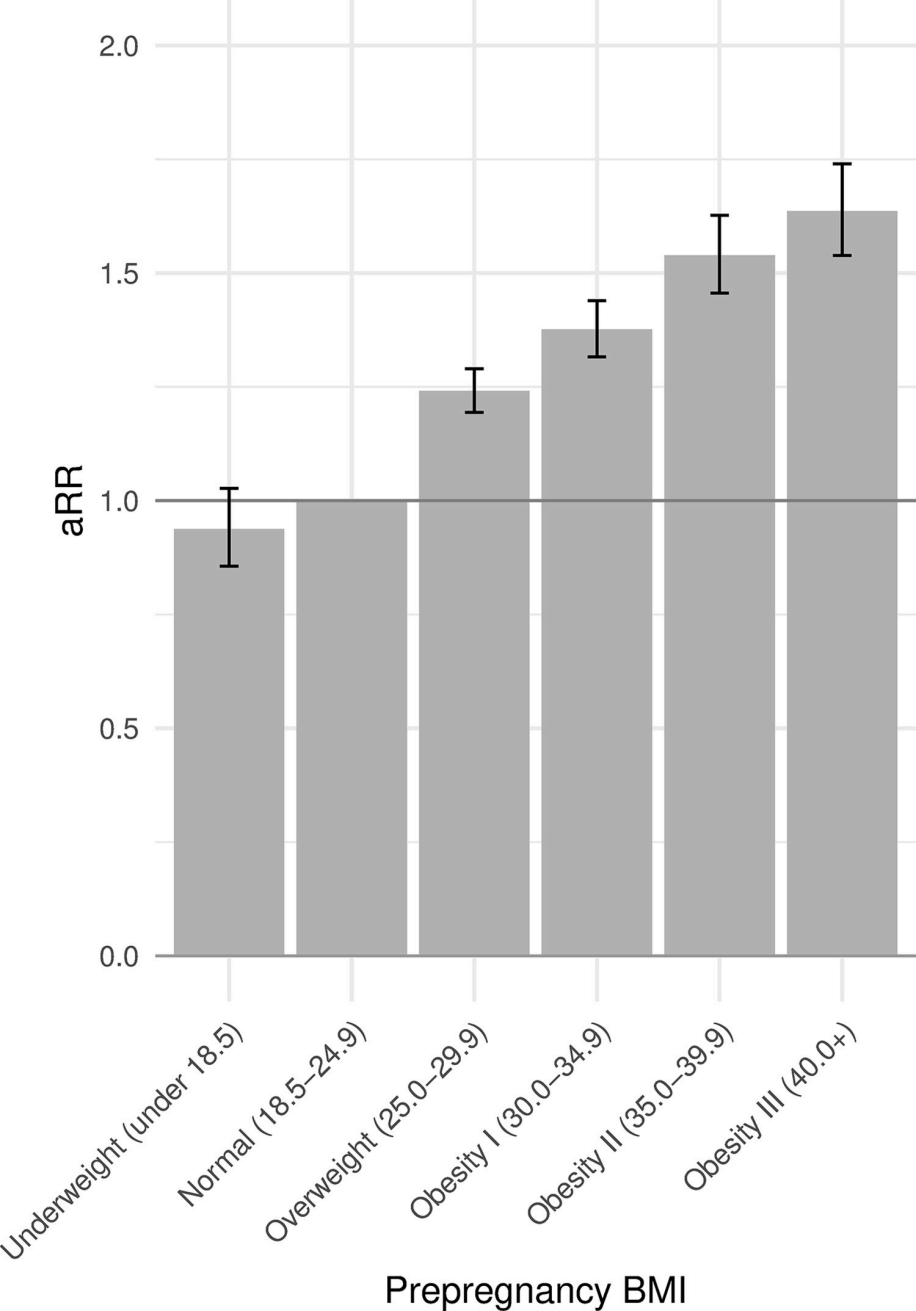
Adjusted relative risk (aRR) of stillbirth by maternal prepregnancy body mass index (BMI). Reference level is normal BMI (18.5–24.9 kg/m^2^).

Table 2 shows results from the second model focusing on medical/obstetric factors and nutritional supplementation, adjusted for sociodemographic variables. Having received no prenatal care was associated with an increased risk of late stillbirth, as was starting prenatal care in the second trimester. Note that risk levels for prenatal care beginning in the third trimester are associated with infant survivorship bias and thus do not reflect the true risk associated with very late onset of prenatal care. Additionally, compared to mothers who have had one previous live birth, first time mothers were at higher risk, and increasing numbers of previous births beyond one were associated monotonically with higher risk of stillbirth. Infants born to mothers who smoked prior to pregnancy and continued smoking were at higher risk of stillbirth compared with infants born to non-smoking mothers, as were infants born to mothers who started smoking during pregnancy, although the numbers in this group were small. On the other hand, risk among mothers who quit smoking by the first trimester was not significantly different than among those who did not smoke. Prepregnancy and gestational hypertension, as well as eclampsia, were associated with increased risk, as were prepregnancy and gestational diabetes, with prepregnancy diabetes markedly so (aRR = 4.54, CI 4.23–4.87). Infertility treatment was also associated with increased risk of stillbirth. Mothers with high BMI were at higher risk of having a stillborn infant compared with mothers who had normal BMI, and there was a clear dose-effect (Fig 3). The greatest risk reduction was associated with participation in the WIC program (aRR = 0.55; CI 0.53–0.57). AUC of this model was 0.73.

**Table 2:** Unadjusted and adjusted relative risk ratios (aRR) over all observations for medical/obstetric factors and nutritional support.

Variable	Live birth N	Stillbirth N	Stillbirth rate per 1000	RR (95% CI)	*Adjusted RR (95% CI)
**Number of previous live births**					
No previous live births	2,620,095	6,703	2.55	**1.24 (1.19, 1.29)**	**1.23 (1.18, 1.28)**
1	2,134,356	4,399	2.06	ref.	ref.
2	1,128,614	3,070	2.71	**1.32 (1.26, 1.38)**	**1.21 (1.16, 1.27)**
3	482,857	1,645	3.40	**1.65 (1.56, 1.75)**	**1.37 (1.30, 1.45)**
4	186,334	812	4.34	**2.11 (1.96, 2.27)**	**1.55 (1.43, 1.67)**
5	76,716	403	5.23	**2.54 (2.29, 2.81)**	**1.66 (1.50, 1.84)**
6	34,634	199	5.71	**2.78 (2.41, 3.20)**	**1.72 (1.49, 1.98)**
7 or more	35,961	272	7.51	**3.65 (3.23, 4.12)**	**1.96 (1.73, 2.21)**
Unknown or not stated	32,590	831			
**Infertility treatment**					
No	6,645,366	17,464	2.62	ref.	ref.
Yes	71,479	240	3.35	**1.28 (1.12, 1.45)**	**1.43 (1.26, 1.62)**
Unknown or not stated	15,312	630			
**Diabetes**					
No diabetes	6,295,229	15,568	2.47	ref.	ref.
Prepregnancy diabetes	52,190	874	16.47	**6.68 (6.24, 7.15)**	**4.54 (4.23, 4.87)**
Gestational diabetes	369,426	1,262	3.40	**1.38 (1.30, 1.46)**	**1.29 (1.21, 1.36)**
Unknown or not stated	15,312	630			
**Hypertension**					
No hypertension	6,271,715	16,054	2.55	ref.	ref.
Hypertension eclampsia	16,121	112	6.90	**2.70 (2.24, 3.25)**	**1.86 (1.55, 2.23)**
Gestational hypertension	343,207	1,300	3.77	**1.48 (1.40, 1.56)**	**1.26 (1.19, 1.33)**
Prepregnancy hypertension	101,114	868	8.51	**3.33 (3.11, 3.57)**	**1.91 (1.78, 2.05)**
**Smoking**					
Non-smoker	5,974,681	14,206	2.37	ref.	ref.
Continued smoking	503,301	2,093	4.14	**1.75 (1.67, 1.83)**	**1.52 (1.45, 1.60)**
Quit smoking	159,264	418	2.62	**1.10 (1.00, 1.22)**	1.09 (0.99, 1.19)
Started smoking	4,094	28	6.79	**2.86 (1.98, 4.15)**	**2.37 (1.65, 3.40)**
Unknown or not stated	90,817	1,589			
**Mother’s prepregnancy BMI**					
Underweight (under 18.5)	240,647	476	1.97	1.01 (0.92, 1.11)	0.94 (0.86, 1.03)
Normal (18.5–24.9)	2,962,807	5,799	1.95	ref.	ref.
Overweight (25.0–29.9)	1,676,696	4,369	2.60	**1.33 (1.28, 1.38)**	**1.24 (1.19, 1.29)**
Obesity I (30.0–34.9)	896,712	2,807	3.12	**1.60 (1.53, 1.67)**	**1.38 (1.32, 1.44)**
Obesity II (35.0–39.9)	428,769	1,600	3.72	**1.90 (1.80, 2.01)**	**1.54 (1.46, 1.63)**
Obesity III (40.0+)	294,929	1,300	4.39	**2.25 (2.12, 2.39)**	**1.64 (1.54, 1.74)**
Unknown or not stated	231,597	1,983			
**Month prenatal care began**					
1st-3rd month	4,993,596	11,519	2.30	ref.	ref.
4th-6th month	1,110,429	3,281	2.95	**1.28 (1.23, 1.33)**	**1.05 (1.01, 1.10)**
7th-final month	290,556	650	2.23	0.97 (0.90, 1.05)	**0.73 (0.67, 0.78)**
No prenatal care	97,293	1,015	10.32	**4.49 (4.21, 4.78)**	**1.99 (1.85, 2.12)**
Unknown or not stated	240,283	1,869			
**Infant sex**					
Female	3,284,130	8,790	2.67	ref.	ref.
Male	3,448,027	9,544	2.76	**1.03 (1.00, 1.06)**	1.03 (1.00, 1.06)
**Nutritional support (WIC)**					
No	3,738,028	9,760	2.60	ref.	ref.
Yes	2,842,549	6,115	2.15	**0.82 (0.80, 0.85)**	**0.55 (0.53, 0.57)**
Unknown or not stated	151,580	2,459			

Number of live births and late stillbirths, late stillbirth rate per 1000 births, and the unadjusted and adjusted relative risks (RRs) and their 95% confidence intervals (CI) for medical/obstetric factors and nutritional supplementation. Numbers in bold indicate category is significantly different from the reference category (ref).

*Adjusted RRs are adjusted for all variables in the table as well as mother’s race, mother’s Hispanic origin, mother’s age, mother’s educational attainment, mother’s nativity, and father’s age.

Table 3 shows the aRRs associated with babies who were SGA and LGA. These risk coefficients were estimated from the third GLM described above which included all risk factors from the first two models as adjustment variables with GROW being the exposure of interest. Babies at both ends of the GROW continuum were at increased risk of stillbirth, with SGA being associated with a marked increase in risk (aRR = 5.43; CI 5.27–5.60). AUC of this third model including GROW was 0.80.

**Table 3:** Unadjusted and adjusted relative risk ratios (aRR) over all observations for adjusted birthweight centile categories.

GROW centile category	Live birth N	Stillbirth N	Stillbirth rate per 1000	RR (95% CI)	*Adjusted RR (95% CI)
AGA	4,785,410	6,893	1.44	ref.	ref.
SGA (10th centile and below)	918,394	8,558	9.23	**6.42 (6.22, 6.63)**	**5.43 (5.27, 5.60)**
LGA (90th centile and above)	1,028,246	2,760	2.68	**1.86 (1.78, 1.95)**	**1.68 (1.61, 1.76)**
Unknown or not stated	107	123			

Number of live births and late stillbirths, late stillbirth rate per 1000 births, and the unadjusted and adjusted relative risks (RRs) and their 95% confidence intervals (CI) for birthweight category. Numbers in bold indicate category is significantly different from the reference category (ref).

*Adjusted RRs are adjusted for mother’s race, mother’s Hispanic origin, mother’s age, mother’s educational attainment, mother’s nativity, father’s age, number of previous live births, infertility treatment, diabetes, hypertension, smoking, BMI, timing of prenatal care onset, infant sex, and WIC.

### Subset analysis: Mothers with a prior live birth

We assessed risk for factors relevant to mothers with at least one prior live birth by restricting analyses to this subset of mothers (4,112,062 live and 11,631 stillbirths; stillbirth rate 2.82/1000 births) and fitting two additional models, as described above. The first model included all risk factors except GROW and prior live birth now dead. Results for interval since last live birth and prior cesarean are shown in Table 4. The modal category for interval since last live birth was 24–35 months, which served as the reference category. After controlling for other risk factors, very short inter-pregnancy intervals and intervals greater than 36 months were associated with increased risk. Prior cesarean section was not significantly associated with stillbirth. AUC of this model was 0.75.

**Table 4:** Results for inter-pregnancy interval and prior cesarean for mothers with at least one prior live birth.

Variable	Live birth N	Stillbirth N	Stillbirth rate per 1000	RR (95% CI)	*Adjusted RR (95% CI)
**Interval since last live birth**					
4–11 months	52,926	310	5.82	**2.86 (2.53, 3.22)**	**2.02 (1.80, 2.27)**
12–17 months	326,570	891	2.72	**1.34 (1.23, 1.45)**	1.08 (1.00, 1.17)
18–23 months	495,606	1,023	2.06	1.01 (0.94, 1.09)	0.95 (0.88, 1.02)
24–35 months	903,323	1,844	2.04	ref.	ref.
36–47 months	590,988	1,334	2.25	**1.11 (1.03, 1.19)**	**1.09 (1.02, 1.16)**
48–59 months	406,410	936	2.30	**1.13 (1.04, 1.22)**	**1.08 (1.00, 1.17)**
60–71 months	295,957	771	2.60	**1.28 (1.17, 1.39)**	**1.21 (1.11, 1.31)**
72 months and over	799,819	2,374	2.96	**1.45 (1.37, 1.54)**	**1.29 (1.21, 1.37)**
Unknown or not stated	240,463	2,148			
**Previous cesarean**					
No	3,099,713	8,468	2.72	ref.	ref.
Yes	1,002,512	2,694	2.68	0.98 (0.94, 1.03)	0.98 (0.94, 1.02)
Unknown or not stated	9,837	469			

Number or live births and late stillbirths, late stillbirth rate per 1000 births and the unadjusted and adjusted relative risks (RR) and their 95% confidence intervals (CI) for inter-birth interval and prior cesarean. Numbers in bold indicate category is significantly different from the reference category (ref).

*Adjusted RRs are adjusted for mother’s race, mother’s Hispanic origin, mother’s age, mother’s educational attainment, mother’s nativity, father’s age, number of previous live births, infertility treatment, diabetes, hypertension, smoking, BMI, timing of prenatal care onset, infant sex, and WIC.

To estimate the remaining risk associated with mothers who have had a prior live birth now dead after controlling for all other risk factors, we fit an additional model with all available risk factors. Results for prior live birth now dead are shown in Table 5. There was a nearly 10-fold increased risk of stillbirth for mothers who have had a prior live-born baby die (aRR 9.69 (CI: 9.25–10.16; Table 5). AUC of the model was 0.83. To assess potential for collider or overadjustment bias, we conducted an additional sensitivity analysis by refitting the model without the GROW variable. The aRR for prior live now dead in the sensitivity analysis was 10.95 (CI: 10.44–11.49). Overall, this suggests that mothers who have had a death from a prior live birth are at considerable residual risk of stillbirth, even after accounting for a large set of known sociodemographic, medical, and obstetric risk factors for the current pregnancy.

**Table 5:** Risk ratios for prior live birth now dead for mothers with at least one prior live birth.

Prior live birth now dead	Live birth N	Stillbirth N	Stillbirth rate per 1000	RR (95% CI)	*Adjusted RR (95% CI)
No	4,000,225	8,315	2.07	ref.	ref.
Yes	79,870	2,562	31.08	**14.98 (14.34, 15.66)**	**9.69 (9.25, 10.16)**
Unknown or not stated	31,967	754			

Number or live births and late stillbirths, late stillbirth rate per 1000 births and the unadjusted and adjusted relative risks (RR) and their 95% confidence intervals (CI) for having had a child from a prior live now dead. Numbers in bold indicate category is significantly different from the reference category (ref).

*Adjusted RRs are adjusted for mother’s race, mother’s Hispanic origin, mother’s age, mother’s educational attainment, mother’s nativity, father’s age, number of previous live births, infertility treatment, diabetes, hypertension, smoking, BMI, timing of prenatal care onset, infant sex, WIC, and GROW.

### Term birth analysis

We performed a sensitivity analysis to exclude potential impacts of pre- and post-term births on risk estimates, focusing only on data for births between 37 and 41 weeks (inclusive) of gestation (5,789,658 live and 6,599 stillbirths; term stillbirth rate = 1.14/1000). Models followed the same stratified approach used for the set using all observations. Results of the three models are shown in S1–S3 Tables. The results of these analyses are largely concordant with those from the models fit on all observations, though some changes in statistical significance reflect a modest reduction in statistical power associated with the reduced number of observations. Model AUCs were 0.64, 0.72, and 0.77, respectively.

### Population attributable fractions (PAFs)

We calculated PAF for those variables that might be modifiable (low/high BMI, WIC, delayed or absent prenatal care, smoking) or could potentially be managed medically (diabetes, hypertension, fetal growth). PAFs consider both the relative risk associated with the factor and the proportion of cases exposed. They thus reflect the proportion of stillbirths that are associated with an exposure after controlling for all other risk factors in the model and, if one assumes causality, the proportion that might be eliminated by removing the exposure [30]. PAFs for the selected exposures are shown in Table 6. Note that the PAF for WIC is negative, indicating its protective association. Because of rounding, some PAFs and confidence interval boundaries show as zero; PAFs whose 95% confidence intervals do not include zero before rounding are therefore marked in bold.

**Table 6:** Population attributable fractions (PAFs) and 95% CIs for selected exposures.

Exposure	PAF (95% CI)
**GROW**	
SGA (10th centile and below)	**0.38 (0.38, 0.38)**
LGA (90th centile and above)	**0.06 (0.06, 0.06)**
**WIC (Yes)**	**-0.28 (-0.30, -0.25)**
**BMI**	
Underweight (under 18.5)	0.00 (-0.01, 0.00)
Overweight (25.0–29.9)	**0.05 (0.04, 0.05)**
Obesity I (30.0–34.9)	**0.04 (0.04, 0.05)**
Obesity II (35.0–39.9)	**0.03 (0.03, 0.03)**
Obesity III (40.0+)	**0.03 (0.02, 0.03)**
**Diabetes**	
Prepregnancy diabetes	**0.04 (0.04, 0.04)**
Gestational diabetes	**0.02 (0.01, 0.02)**
**Prenatal Care**	
4th-6th month	**0.01 (0.00, 0.02)**
No prenatal care	**0.03 (0.02, 0.03)**
**Hypertension**	
Hypertension eclampsia	**0.00 (0.00, 0.00)**
Gestational hypertension	**0.01 (0.01, 0.02)**
Prepregnancy hypertension	**0.02 (0.02, 0.02)**
**Smoking**	
Continued smoking	**0.04 (0.04, 0.04)**
Quit smoking	0.00 (0.00, 0.00)
Started smoking	**0.00 (0.00, 0.00)**

CIs for PAFs were obtained using bootstrap methods. PAFs with CIs that do not include zero before rounding are indicated in bold.

The exposures with the largest attributable fractions were SGA (38%) and mothers who had high BMI (15% cumulatively across BMI levels of overweight and higher). Prepregnancy and gestational diabetes were associated with 4% and 2% of stillbirths respectively, while prepregnancy and gestational hypertension were associated with approximately 2% and 1% of stillbirths respectively. Exposure to smoking was associated with approximately 4% of stillbirths. This was predominantly among mothers who continued to smoke during pregnancy, as, despite being at markedly higher risk, the number of mothers who began smoking during pregnancy was very low, leading to a low PAF. Delayed or absent prenatal care accounted for around 4% of stillbirths cumulatively across factor levels. Receipt of supplemental nutrition benefits was associated with approximately a 28% reduction in the overall number of stillbirths after controlling for other variables.

## Discussion

This study of late stillbirth provides evidence for factors related to late stillbirth risk in the US. This includes updated population-based risk estimates for some previously documented factors and a US-level baseline estimate for risk reduction associated with receipt of nutritional support (WIC) benefits, as well as identifying modifiable or manageable factors that contribute to a large proportion of late stillbirths.

The importance of social determinants of health is well known and these determinants are reiterated in this study. Black and Native Hawaiian and Other Pacific Islander mothers were at higher risk of late stillbirth compared with White mothers. Mothers with low educational attainment showed higher risk of stillbirth, as did mothers aged 35 or older. Our study aligned with previous work showing the optimal interpregnancy interval [32]. Both short (<12 months) and longer (>36 months) intervals between pregnancies were associated with an increased risk of stillbirth. Also consistent with previous research, infertility treatment was associated with late stillbirth [33], although only 1.1% of women reported receiving treatment.

Medical problems such as diabetes and hypertension were associated with an increased risk of late stillbirth. Although the PAFs for these factors showed that only 3–6% of stillbirths could be attributed to each of them, they are nonetheless medically manageable and continued emphasis on proactive treatment could reduce the likelihood of stillbirth.

High maternal BMI was identified as a major risk factor. There was a clear gradient of increasing late stillbirth risk associated with increasing BMI. Given the percentage of pregnant women have BMIs in the overweight and obesity ranges (25.9% and 24.5% respectively in our dataset), a large proportion of stillbirths can be attributed to this risk factor: 15% cumulatively across high BMI categories after adjustment for covariates in our PAF analysis. The increasing obesity epidemic in the US and around the world will result in this factor being of increasing concern [34].

Over 10% of women in our dataset reported smoking, but almost a quarter of women who smoked prior to pregnancy did not smoke during any pregnancy trimester (24.0%). These women were not at significantly higher risk of late stillbirth compared to mothers who smoked neither before nor during pregnancy. This is an important public health message, namely that quitting smoking prior to pregnancy may reduce stillbirth risk. Smoking data should be treated with some caution, however, as the CDC data rely on self-reports; objective measurements of smoking were not available. Furthermore, almost all pregnant women know that smoking is harmful and may under report the amount smoked or report quitting when they have not [35]. Therefore, our risk estimates for smoking may be an underestimate and should be interpreted as lower-bounds.

Another public health message is the importance of prenatal care. Prenatal care began in the first trimester in 77.7% of all pregnancies; however, delaying prenatal care until the second trimester was associated with a 5% increase in the risk of stillbirth, and missing prenatal care completely was associated with a near-doubling of risk. Our PAF results suggest that approximately 3% of stillbirths were associated with delayed or missing prenatal care. Encouraging mothers to seek prenatal care at the start of pregnancy might therefore reduce this portion of stillbirth burden.

Using customized growth centiles, 12.0% of births were categorized as SGA. SGA was associated with over a five-fold increased risk of late stillbirth, suggesting that SGA contributes substantially to the overall burden of stillbirth in the US, 38% according to our covariate-adjusted PAF analysis. It is not known whether the growth restriction was recognized before birth for mothers in our dataset and whether that influenced the risk. Despite fetal growth monitoring, definitively diagnosing growth restrictions remains difficult, such that fetal growth restriction is missed in pregnancy in about of a third of births [36]. The dataset does not record whether or not fetal growth restriction was recognized prior to delivery, nor whether or not uterine artery doppler was undertaken [37]. Furthermore, there is no proven treatment for growth restriction, so management must include a balance of the risks of intrauterine chronic hypoxia with iatrogenic preterm delivery and its associated risks [38].

One potential caveat to our finding regarding SGA stems from potential ambiguity around the date of fetal death. The exact date of fetal death is mostly unknown and there is some delay between fetal death and diagnosis/delivery; thus, assessment of gestational age at death is subject to some error [39]. If date of delivery is used as the measure of gestational age in cases of fetal death, as is done in the CDC fetal death data files, then gestational age will be overestimated resulting in an overestimate of SGA and underestimate of LGA and their respective risks [40]. The GROW algorithm subtracts 2 days from the gestational age at birth for stillborn infants to account for this. We acknowledge that this might not be sufficient for some cases. However, for pregnancies that have reached term, pregnant women are assessed weekly, so that the interval between the fetus being noted to be alive and now dead is a maximum of 7 days (5 days after correction by GROW, with a lower bound of -1 days after correction). When we restricted the analysis to term stillbirths, no trade-off between SGA and LGA categorizations was found compared to the full analysis, and the magnitude of risk for both SGA and LGA was similar between the full and term birth analyses. This lends confidence to our risk estimates for these variables.

One of the most striking and unexpected findings in the study was the 45% reduction in risk of late stillbirth for recipients of WIC in pregnancy. WIC is the Special Supplemental Nutrition Program for Women, Infants, and Children which is an American federal assistance program of the Food and Nutrition Service of the US Department of Agriculture for healthcare and nutrition of low-income pregnant women and new mothers. Specific WIC benefits vary by jurisdiction, but they include a mix of nutrition education, referrals to healthcare and other social services, vouchers for food, and direct food delivery [15,41]. WIC has been shown to reduce infant mortality and improve a number of health outcomes among live-born infants [10–15,42]. However, we are aware of no other existing US population-level research on the association between WIC participation and stillbirth risk, though there are some smaller region-specific studies of relevance. One study using California Medi-Cal data found a reduction in risk of perinatal death (stillbirth and early neonatal death) between 22–31% for gestational periods between 29 and 43 weeks, which approximately aligns with gestational ages in our study [13]. Two additional smaller observational studies also found some evidence for a protective role of WIC in stillbirth, at least in certain sub-groups of mothers (e.g., Black mothers and mothers with lower educational attainment) [11,43]. Further work is needed to validate whether these same interactions between sociodemographic variables and WIC receipt hold in US-level data. Additionally, a Cochrane review of randomized controlled trials for effects of direct dietary supplementation in perinatal outcomes found that balanced energy and protein supplementation for mothers reduced stillbirth risk (RR = 0.60; CI: 0.39–0.94). These reports, in concert with our study, suggest a promising role for nutritional supplementation in reducing stillbirth risk.

In our study, 42% of pregnant women reported receiving WIC. As this study is observational one cannot assume causality, but receipt of WIC was associated with a large reduction in overall stillbirth burden– 28% in our adjusted analysis. While our stillbirth finding is concordant with previous studies on other fetal health outcomes, it comes with a number of caveats: we were unable to determine specifically which mothers were eligible for WIC in our dataset so that coverage rates for the benefit among eligible mothers are unknown and selection biases could not be ruled out; we were unable to determine date of WIC enrollment for those who did receive WIC benefits and thus could not determine duration of WIC benefits receipt; and we do not know the density of mothers’ participation in the program (e.g., number of food packages received). Our initial US population-level finding is suggestive of a protective benefit for WIC participation, though we acknowledge that after accounting for the above limitations effect size estimates may change. This finding is concordant with other smaller observational studies and randomized controlled trials on nutritional supplementation, though we take it to be preliminary and hypothesis-generating for future follow-up research.

Our study has several limitations. First, our findings regarding WIC are subject to the limitations described above. Second, many studies exclude congenital anomalies, but this information was not available for our analyses. However, many stillbirths are not fully examined, so the cause including congenital anomalies might be missed. Third, the lack of data concerning fetal movements and maternal sleep means that these risk factors could not be examined [44]. Fourth, we do not have information on prior adverse pregnancy outcomes for fetal deaths, so this information could not be included in the analyses. Additionally, there may be some inaccuracies in physician-reported data on the US Standard Report of Fetal Death or US Standard Certificate of Live Birth [45]. These inaccuracies would be propagated to the CDC Vital Statistics datasets which we used here, and which are also commonly used by others doing population-level research on perinatal and infant health outcomes in the US. However, limiting our set of observations to those from areas identified by the CDC as having high reporting quality mitigates some concerns.

There may be additional bias in some remaining self-reported risk factors. For example, as mentioned above, smoking has been documented to be under-reported by mothers, and the CDC Vital Statistics data may reflect this. Nonetheless, we note that the aRRs for continuing smoking from our models are similar to those reported in prior meta-analyses for high-income countries [3]. However, all other previously published similar risk factor research and related meta-analyses would reflect this same under-reporting bias. The risk estimates and PAFs for smoking reported by us and others in the literature should therefore be interpreted as lower bounds on the true risk and attributable fractions.

In conclusion, while this study emphasizes the importance of social determinants on stillbirth risk [46], several of our major findings on potentially modifiable risk factors can be of use to medical professionals and policymakers, even if further confirmation with randomized controlled trials is not feasible. In the areas of family planning and medical care, stillbirth burden may be reduced if mothers have their children before age 35, avoid very short or very long interpregnancy intervals, seek prenatal care early, manage diabetes and hypertension, and quit smoking. Future work should additionally consider interactions among these risk factors, as mothers with different combinations of risk factors may benefit from different interventions.

Several of our findings can also be of use to policymakers. Solutions for the obesity epidemic are urgently needed, as reductions in obesity could lead to a substantial reduction in stillbirth rates. Also, programs to improve the identification and management of fetal growth restriction warrant support. We additionally found that participation in the WIC program was associated with reduced stillbirth risk; expanding eligibility for WIC or at least promoting participation among those already eligible might further reduce stillbirth rates. However, this finding for WIC is preliminary, subject to several caveats described above, and deserves more in-depth follow-up.

## Supporting information

S1 Table Unadjusted and adjusted relative risk ratios (aRR) for term births for sociodemographic factors. (XLSX)

S2 Table Unadjusted and adjusted relative risk ratios (aRR) for term births for medical/obstetric factors and nutritional support. (XLSX)

S3 Table Unadjusted and adjusted relative risk ratios (aRR) over all observations for adjusted birthweight centile categories. (XLSX)
